# SIMSI-Transfer: Software-Assisted Reduction of Missing Values in Phosphoproteomic and Proteomic Isobaric Labeling Data Using Tandem Mass Spectrum Clustering

**DOI:** 10.1016/j.mcpro.2022.100238

**Published:** 2022-04-21

**Authors:** Firas Hamood, Florian P. Bayer, Mathias Wilhelm, Bernhard Kuster, Matthew The

**Affiliations:** Chair of Proteomics and Bioanalytics, Technical University of Munich, Freising, Germany

**Keywords:** isobaric labeling, missing values, spectrum clustering, identification transfer, phosphoproteomics, CPTAC, Clinical Proteomic Tumor Analysis Consortium, FDR, false discovery rate, FP, full proteome, IMBR, isobaric match-between-runs, LFQ, label-free quantification, MBR, match-between-runs, MS, mass spectra, PP, phosphoproteome, PSM, peptide-spectrum match, SIMSI-Transfer, Similarity-based Isobaric MS2 Identification Transfer, TMT, tandem mass tag

## Abstract

Isobaric stable isotope labeling techniques such as tandem mass tags (TMTs) have become popular in proteomics because they enable the relative quantification of proteins with high precision from up to 18 samples in a single experiment. While missing values in peptide quantification are rare in a single TMT experiment, they rapidly increase when combining multiple TMT experiments. As the field moves toward analyzing ever higher numbers of samples, tools that reduce missing values also become more important for analyzing TMT datasets. To this end, we developed SIMSI-Transfer (Similarity-based Isobaric Mass Spectra 2 [MS2] Identification Transfer), a software tool that extends our previously developed software MaRaCluster (© Matthew The) by clustering similar tandem MS2 from multiple TMT experiments. SIMSI-Transfer is based on the assumption that similarity-clustered MS2 spectra represent the same peptide. Therefore, peptide identifications made by database searching in one TMT batch can be transferred to another TMT batch in which the same peptide was fragmented but not identified. To assess the validity of this approach, we tested SIMSI-Transfer on masked search engine identification results and recovered >80% of the masked identifications while controlling errors in the transfer procedure to below 1% false discovery rate. Applying SIMSI-Transfer to six published full proteome and phosphoproteome datasets from the Clinical Proteomic Tumor Analysis Consortium led to an increase of 26 to 45% of identified MS2 spectra with TMT quantifications. This significantly decreased the number of missing values across batches and, in turn, increased the number of peptides and proteins identified in all TMT batches by 43 to 56% and 13 to 16%, respectively.

## Highlights

• Spectrum clustering enables peptide identification transfer between LC–MS/MS runs.
• The SIMSI pipeline supports processing full proteome and phosphoproteome data.
• SIMSI increases the number of quantifiable PSMs by 26 to 45%.
• SIMSI reduces missing values in multibatch TMT labeling experiments by up to 21%.

## Graphical Abstract

**Figure:**
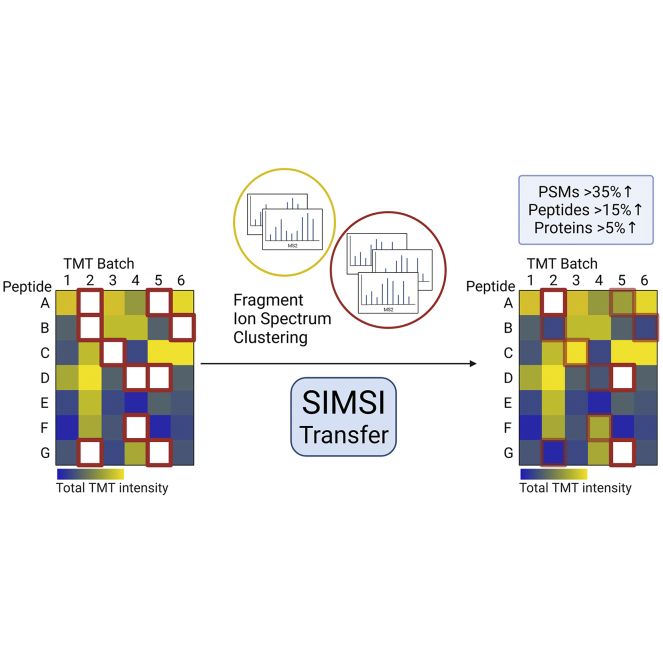

## In Brief

We developed a tool, SIMSI-Transfer, which reduces missing values in experiments that consist of multiple TMT batches. It works by transferring peptide identifications from one TMT batch to another based on the similarity of tandem mass spectra. SIMSI-Transfer increases the number of quantifiable peptide-spectrum matches by 30% with a concomitant reduction in missing values between TMT batches and is applicable to both full proteome and phosphoproteome experiments. SIMSI is available on GitHub.

Isobaric stable isotope labeling techniques such as tandem mass tags (TMTs) are frequently used for proteome profiling of large patient cohorts, exemplified by several projects of the Clinical Proteomic Tumor Analysis Consortium (CPTAC) (1). This is because the multiplexing capability of TMT offers substantial sample throughput (up to 18) as well as consistent and precise relative peptide and protein quantification within one multiplexed TMT experiment. It is also more robust than label-free quantification (LFQ) against variations in the performance of the chromatographic separation system because quantification is performed on level 2 or 3 tandem mass spectra (MS2 or MS3). This, in turn, simplifies method transfer, for example for multi-center clinical projects. The downside of using TMT labeling is that quantitative accuracy and dynamic range are typically poorer compared with LFQ because of the well-documented effect of ratio compression (2). A further issue arises when combining multiple TMT experiments (*i.e.*, batches) into one analysis. Because the set of identified and quantified peptides is not necessarily the same in each TMT batch, the amount of missing data (peptide identification and quantifications) becomes an increasing concern the more TMT batches are combined. For instance, less than half of all peptides were quantified in all the 24 batches analyzed in a study of induced pluripotent stem cell lines, and a similar observation was made in the analysis of 28 batches of ovarian cancer samples (3, 4). These missing values pose challenges for downstream analysis tools that often require full data matrices to work (4).

For LFQ, the reduction of missing values has already received considerable attention. The most popular approach is to find corresponding MS1 features between samples (*i.e.*, peptide precursor ions of the same mass-to-charge ratio and retention time), for example, using the match-between-runs (MBR) procedure in MaxQuant (© Max-Planck-Institute of Biochemistry) (5, 6, 7). This is an attractive approach because it does not require an MS2 spectrum for that peptide in each experiment. The downside is that, because there is no MS2 spectrum that can be used to verify the match, some uncertainty remains as to how correct the assignments are (8). As an alternative, MS2 spectrum similarity clustering has been proposed to reduce missing values in LFQ experiments (9). In this approach, MS2 spectra are compared to each other using a distance metric, and highly similar spectra are grouped together into clusters. The underlying assumption is that all MS2 spectra in one cluster represent the same peptide precursor ion (10, 11, 12). If so, the peptide-spectrum match (PSM) of identified MS2 spectra could be transferred to unidentified spectra in the same cluster, since they represent the same precursor peptide. These spectra can originate from different experiments, in which, for example, the quality of the MS2 spectrum was insufficient for identification by database searching.

For isobaric labeling, the missing value problem has only recently begun to receive substantial consideration (3, 4, 13). For instance, Yu *et al.* (13) applied the MBR idea to TMT data (termed isobaric match-between-runs [IMBR]) by matching MS1 features as in the original MBR approach but in addition extracting the quantification information from the MS2 or MS3 spectra. While conceptually straightforward, this approach does not guarantee that the assigned MS2 spectrum actually belongs to the matched precursor ion, especially when dealing with samples of high complexity where MS1 isotope patterns frequently overlap (14, 15). As in the case of LFQ data, MS2 spectrum clustering can also be used for TMT data. Compared with IMBR, spectral clustering is less sensitive to the issue of overlapping MS1 isotope patterns as the transfers of identifications are based on MS2 spectrum similarity rather than similar retention times and mass-to-charge ratios only. MaRaCluster (© Matthew The) (11) is one such spectrum clustering tool that showed competitive performance over others (16, 17) and can also be used for TMT data. However, MaRaCluster has not yet been able to combine data from several TMT batches for the purpose of reducing missing quantification values.

Here, we present SIMSI-Transfer (Similarity-based Isobaric MS2 Identification Transfer), a pipeline that extends the functionality of MaRaCluster by clustering MS2 spectra of isobaric labeling experiments and transferring identifications based on those clusters between TMT experiments. Benchmarking the pipeline using a data masking approach demonstrated high recall (85% for full proteome and 81% for phosphoproteome data) and a false discovery rate (FDR) below 1% using the recommended parameters. The application of this pipeline to the reanalysis of six published CPTAC datasets increased the number of PSMs by up to 45%. This corresponded to an increase in the proportion of quantified peptides and proteins found in all batches by up to 56% and 16%, respectively, thereby reducing missing values on both levels. SIMI-Transfer is programmed in Python and comes with a graphical user interface that allows users to apply SIMSI-Transfer to the analysis of MaxQuant results.

## Experimental Procedures

### Datasets

We used three public studies provided by CPTAC (18, 19, 20) for the evaluation of the SIMSI-Transfer pipeline. Each study consists of a full proteome and a phosphoproteome dataset. The datasets were downloaded from the CPTAC data portal in May 2021 (https://cptac-data-portal.georgetown.edu/datasets) utilizing the IBM Aspera client.

The study by Dou *et al.* (18) consists of endometrial carcinoma samples from 95 patients, measured in 17 batches. It is accessible *via* the Proteomic Data Commons identifiers PDC000125 (full proteome, FP) and PDC000126 (phosphoproteome, PP). The study by Gillette *et al.* (19) covers 111 lung adenocarcinoma samples in 25 batches and is accessible *via* the identifiers PDC000153 (FP) and PDC000149 (PP). Finally, the study by Krug *et al.* (20) consists of breast cancer samples from 134 patients measured in a total of 17 batches and is accessible using the identifiers PDC000120 (FP) and PDC000121 (PP). All datasets used TMT10plex for labeling, with the first nine channels containing patient samples and the last channel containing a common reference sample (“bridge channel”). Samples from the same patient were frequently measured multiple times in different batches or in different TMT channels of the same batch.

The full proteome samples were deep fractionated into 24 (Dou *et al.*) or 25 (Gillette *et al.* and Krug *et al.*) fractions and contain between 16 and 25 million MS2 spectra per dataset, whereas the phosphoproteome samples were fractionated in 12 (Dou *et al.*) or 13 (Gillette *et al.* and Krug *et al.*) fractions and contain between 9 and 14 million MS2 spectra per dataset.

For analyzing the effect of transferred PSMs on peptide- and protein-level quantification, we used a TMT 11plex proteome mixture dataset by Thompson *et al.* (21). It consists of human and yeast proteome samples distributed across three TMT 11plex batches with three different yeast-to-human ratio patterns, which were generated by adding different amounts of yeast cell lysate to a fixed amount of HeLa cell lysate. The dataset is accessible from the ProteomeXchange Consortium *via* the PRIDE identifier PXD014750.

### MaxQuant

Each dataset was analyzed using MaxQuant, version 1.6.17.0 (22). Searches were performed using TMT 10plex default settings, and trypsin with allowed cleavages before proline residues was used for *in silico* digestion with a maximum of two allowed missed cleavages. Cysteine carbamidomethylation was set as a fixed modification, and methionine oxidations as well as N-terminal acetylations were set as variable modifications. For the phosphoproteome data, phosphorylation of serine, threonine, and tyrosine was added as variable modifications. Mass tolerances of 20 ppm for precursor ions during the first search, 4.5 ppm during the main search, and 20 ppm for MS/MS fragment ions were applied. An up-to-date reference proteome from UniProt was downloaded on August 20, 2020 and used for all database searches, and the results were filtered for 1% peptide and 1% protein FDR. The msms.txt as well as the msmsScans.txt result files were used for further data processing.

### MaxQuant IMBR Search

To compare our transferring pipeline with the IMBR algorithm provided within MaxQuant, the Dou *et al.* cohort dataset was also analyzed with IMBR enabled using the default IMBR parameters (match time window of 0.7 min and alignment time window of 20 min). All other parameters were kept identical to the non-MBR search. The resulting msmsScans.txt file was used to identify and determine the number of matched MS2 scans.

### MaRaCluster

Similarity clustering of MS2 spectra was performed using MaRaCluster, version 1.01.1. The CPTAC studies provided .mzML files, which can be used directly as input to MaRaCluster. MaRaCluster returns clustering results for various thresholds that correspond to the –log10(*p-*value) threshold for complete linkage hierarchical clustering. In the current study, results for the six default thresholds -5, -10, -15, -20, -25, and -30 (referred to as p5, p10, p15, p20, p25, and p30 throughout this article) were systematically evaluated to find a good compromise between maximizing the number of transfers and keeping errors at an acceptable level. p5 denotes the lowest clustering stringency, whereas p30 represents the most stringent clustering. MaRaCluster outputs one cluster result file for each of the thresholds.

### SIMSI-Transfer Algorithm

The main algorithm can be divided into three steps:

1. Table merging: MaRaCluster’s output is merged with columns of the MaxQuant msms.txt file based on the MS2 scan number and input file name. The resulting table contains all MS2 scans together with the cluster-ID assigned by MaRaCluster and MaxQuant identification information for each identified spectrum filtered for 1% peptide and protein FDR.
2. Cluster categorization: Clusters are categorized based on the peptide sequence identified by their constituting MS2 spectra:
  a. Singleton clusters contain just a single spectrum (identified or unidentified).
  b. Unanimously identified clusters contain spectra that all identified the same peptide sequence.
  c. Fully unidentified clusters exclusively contain spectra that could not be identified.
  d. Ambiguous clusters contain identified spectra with different unmodified sequences.
  e. Transferable clusters contain both identified and unidentified spectra. All identified spectra have the same modified and unmodified sequence.
  f. PTM-isomeric clusters contain both identified and unidentified spectra. All identified spectra have the unmodified sequence, but they differ in their modification sites.
3. Identification transfer: Using all clusters flagged as “transferable” (class [e] clusters), the peptide sequence is transferred from the identified “donor” spectra to the unidentified “acceptor” spectra alongside with information from MaxQuant, such as the associated protein information. Clusters flagged as “PTM-isomeric” (class [f] clusters) are also used for transferring, but no single modified sequence is transferred (see section “Handling Of Clusters With Positional PTM-Isomers”).

Class (a), (b), and (c), and (d) clusters are not used in this step because no identification transfers are possible in those cases.

The resulting file resembles a MaxQuant msms.txt output file to which columns have been added that relay the results of the SIMSI-Transfer, notably MaRaCluster’s cluster-ID, cluster category according to the aforementioned classification, and the identification type (“direct” for directly identified by MaxQuant or “transfer” for transferred by our pipeline).

### Handling of Clusters with Positional PTM-Isomers

Ambiguous clusters are not suitable for transferring peptide identifications since it is unclear which identification should be transferred. For modified peptides, however, MS2 spectrum clustering methods such as MaRaCluster tend to cluster positional PTM-isomers, for example, phosphoisomers, especially when multiple potential sites are in close proximity (Table 1). In these clusters, which are flagged as “PTM-isomeric” rather than “ambiguous,” the unmodified sequences in the cluster are identical, but the modified sequences can differ concerning the position of the modification (ambiguous localization and confident identification). Since the similarity information gained from such clusters is still highly valuable, we transfer PTM-peptides based on the unmodified peptide sequence ignoring the localization of the PTM for this step. The output file then contains all potential localizations of modified peptides observed in a cluster. Thus, we give the user the flexibility to handle ambiguous localization of PTMs in their preferred way while transferring as much information as possible. For all results presented in this study, metrics were calculated based on unmodified sequences unless otherwise stated.

**Table 1:** Example for handling clusters that contain positional isomers of phosphopeptides

RawFile	ScanID	ClusterID	Sequence	Modified sequence	Protein	ID
rawfile1.raw	5582	75	RASPSPRAA	RApSPSPRAA	Serine/arginine repetitive matrix protein 1	Direct
rawfile1.raw	5588	75	RASPSPRAA	RASPpSPRAA	Serine/arginine repetitive matrix protein 1	Direct
rawfile1.raw	5602	75	—	—	—	—
rawfile2.raw	6025	75	RASPSPRAA	RApSPSPRAA	Serine/arginine repetitive matrix protein 1	Direct
rawfile3.raw	6033	75	—	—	—	—

RawFile	ScanID	ClusterID	Sequence	Modified sequence	Protein	ID
rawfile1.raw	5582	75	RASPSPRAA	RApSPSPRAA	Serine/arginine repetitive matrix protein 1	Direct
rawfile1.raw	5588	75	RASPSPRAA	RASPpSPRAA	Serine/arginine repetitive matrix protein 1	Direct
rawfile1.raw	5602	75	RASPSPRAA	RASPSPRAA.1.p3/p5	Serine/arginine repetitive matrix protein 1	Transf.
rawfile2.raw	6025	75	RASPSPRAA	RApSPSPRAA	Serine/arginine repetitive matrix protein 1	Direct
rawfile3.raw	6033	75	RASPSPRAA	RASPSPRAA.1.p3/p5	Serine/arginine repetitive matrix protein 1	Transf.

Not all output columns are shown here for better readability. *Top*, cluster before transfers. The cluster shown here consists of five MS2 spectra from three different experiments. The same unmodified sequence was identified in experiment 1 (spectra 5582 and 5588) and experiment 2 (spectrum 6025), and two spectra in the cluster were not identified by MaxQuant (experiment 1, spectrum 5602; experiment 3, spectrum 6033). The identifications differ in the phosphorylation sites, as marked in *red*, and the cluster therefore is a class (f) post-translational modification (PTM)-isomeric cluster. *Bottom*, because the exact modified sequence is not known for the whole cluster, SIMSI-Transfer transfers the unmodified peptide sequence with a localization flag shown in *green* as well as the protein information. The flag consists of the total number of modifications (in this case, one PTM) and all observed modification sites (in this case, phosphorylation sites on position 3 or 5 of the sequence). Finally, the ID column indicates that the spectra were identified by SIMSI-Transfer as opposed to a direct identification of the search engine.

### Masking Analysis

To assess the precision and recall of the SIMSI-Transfer pipeline, we masked the identifications of a portion (5%, 10%, 20%, or 50%) of the spectra identified by MaxQuant in each result file before applying our pipeline. These masked spectra, for which the ground truth identification is known, become available as transfer acceptors for the SIMSI-Transfer pipeline and can in turn be used to assess the precision and recall of the process. When treating the reidentification of spectra as a multiclass classification problem with each peptide sequence being a class, precision and recall can be calculated as microaverages of the successful and unsuccessful identifications (23). For each peptide sequence *i*, precision and recall can be calculated as:

$$\text{Precision}\left(i\right)=\;\frac{TP_{i}}{TP_{i}+FP_{i}}\;=\;\frac{\text{Correct transfers of spectra containing peptide }i}{\text{All transfers of spectra as peptide }i}$$

$$\text{Recall}\left(i\right)=\;\frac{TP_{i}}{TP_{i}+FN_{i}}\;=\frac{\text{Correct transfers of spectra containing peptide }i}{\text{All transfers of spectra containing peptide }\;i}$$

where TP, FP, and FN refer to the true positive, false positive, and false negative, respectively. The overall precision and recall can then be calculated as the microaverage across all peptide sequences:

$$\text{Precision}=\;\frac{\sum_{i=1}^{n}TP_{i}}{\sum_{i=1}^{n}TP_{i}+\sum_{i=1}^{n}FP_{i}}\;=\;\frac{\sum \text{Correct transfers}}{\sum \text{Transfers}}$$

$$\text{Recall}=\;\frac{\sum_{i=1}^{n}TP_{i}}{\sum_{i=1}^{n}TP_{i}+\sum_{i=1}^{n}FN_{i}}\;=\;\frac{\sum \text{Correct transfers}}{\sum \text{Masked spectra}}$$

The FDR is then calculated as:

FDR=1−Precision

Precision and recall were calculated for each clustering stringency and compared to each other to find the ideal clustering stringency parameter.

## Results

### SIMSI-Transfer Workflow and Evaluated Datasets

**Fig. 1:**
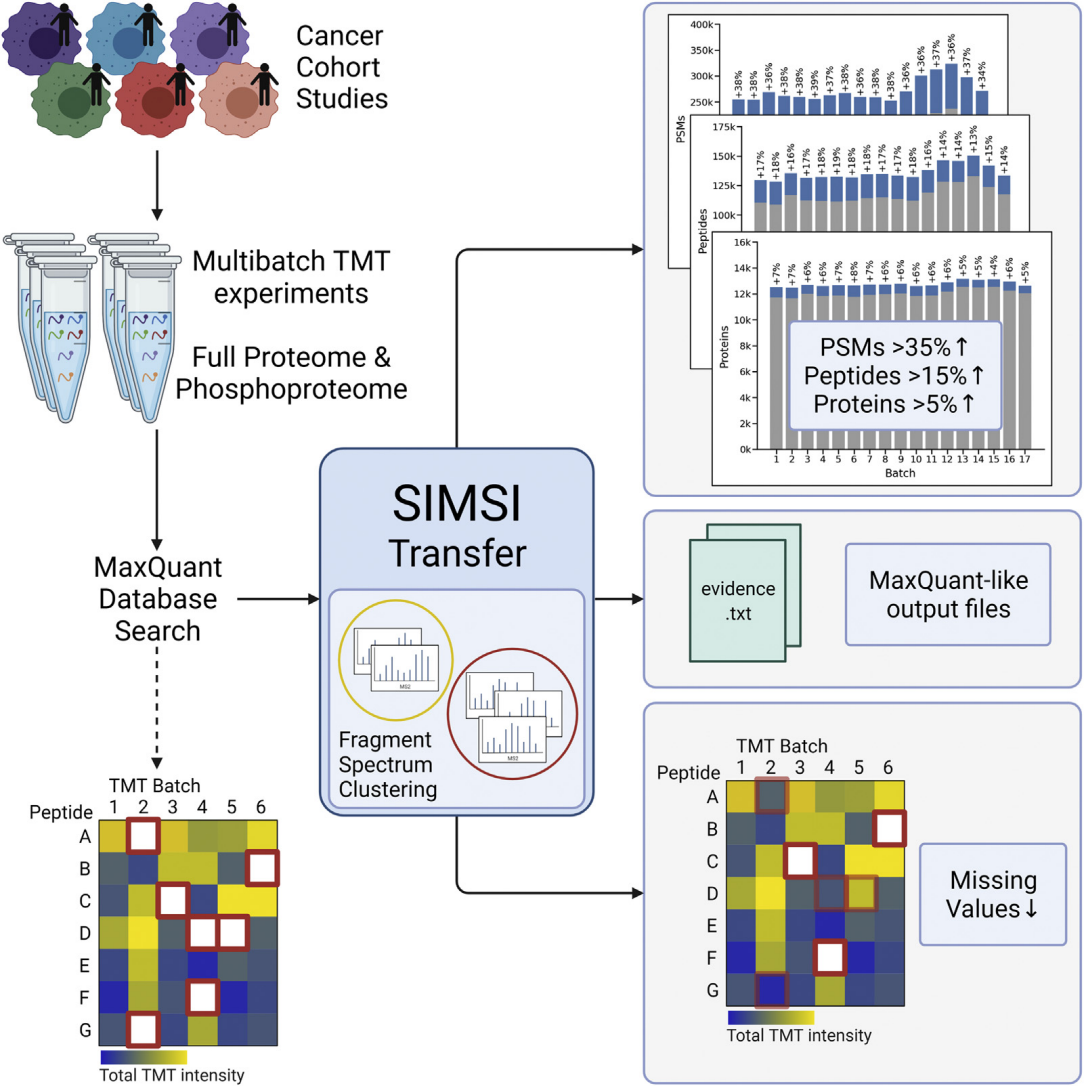
**Summary of the SIMSI-Transfer workflow.** SIMSI-Transfer uses fragment spectrum clustering to transfer identities across TMT batches, resulting in more PSMs, peptides, and proteins, and reduced missing values. *Red squares* in the matrices denote missing values. PSM, peptide-spectrum match; SIMSI-Transfer, Similarity-based Isobaric MS2 Identification Transfer; TMT, tandem mass tag.

SIMSI-Transfer is a tool for reducing missing values in database search results of multibatch isobaric labeling experiments (Fig. 1). After processing the raw MS files with MaxQuant, SIMSI-Transfer applies MS2 spectrum clustering to the raw MS files and transfers peptide identifications across TMT batches by combining the MaxQuant and clustering results. As more peptides are identified in more batches, the number of missing values decreases. We evaluated six different clustering stringencies for MaRaCluster, with p5 being the least stringent clustering and p30 being the most stringent clustering threshold. The result files of the SIMSI-Transfer pipeline are structured similarly to the ones provided by MaxQuant, making them compatible with downstream data analysis pipelines established for MaxQuant output files. We analyzed three CPTAC studies involving three different cancer types (endometrial carcinoma, lung adenocarcinoma, and breast cancer). A summary of these datasets is presented in Table 2. All studies contain both full and phosphoproteome measurements and comprise multiple TMT10plex batches (between 17 and 25 batches), which were in addition fractionated by basic pH reversed-phase liquid chromatography. This resulted in 16.3 to 24.5 million MS2 spectra for the full proteome samples and 8.6 to 14.1 million MS2 spectra for the phosphoproteome samples. Processing these datasets with MaxQuant alone resulted in 16.3 million PSMs and identification rates of 21 to 22% for the full proteome samples and 12 to 15% for the phosphoproteome samples, respectively.

**Table 2:** Summary of the datasets used in this study

Name of dataset	Cancer type	Patients	Batches	Fractions	Total MS2 spectra	MaxQuant PSMs
Dou *et al.*	Endometrial carcinoma	95	FP & PP: 17	FP: 24	16.3 M	3.38 M
				PP: 12	8.63 M	1.29 M
Gillette *et al.*	Lung adenocarcinoma	111	FP & PP: 25	FP: 25	24.5 M	5.41 M
				PP: 13	14.1 M	1.67 M
Krug *et al.*	Breast cancer	134	FP & PP: 17	FP: 25	17.2 M	3.53 M
				PP: 13	9.96 M	1.20 M

Abbreviations: FP, full proteome; PP, phosphoproteome.

### Evaluating the Performance of SIMSI-Transfer

**Fig. 2:**
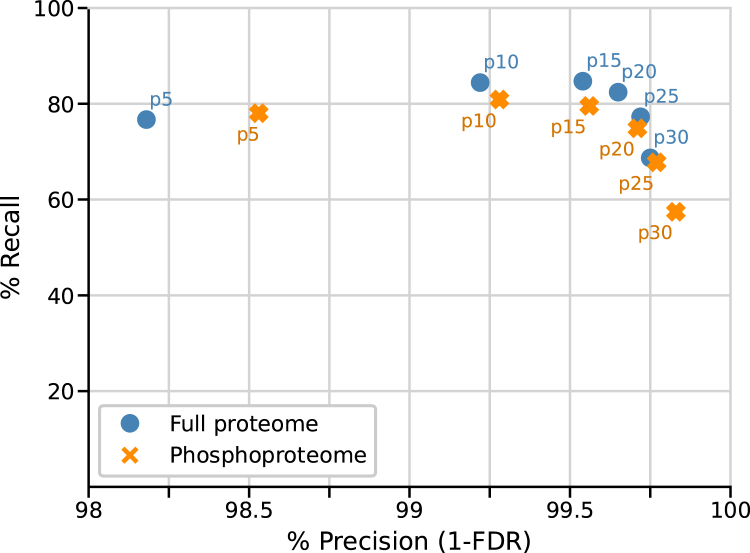
**Precision-recall plot assessing the performance of SIMSI-Transfer illustrated by masking 10% of the Dou *et al*. dataset.** The full proteome and phosphoproteome dataset show a precision of >99% at a clustering stringency of p10 or higher. SIMSI-Transfer, Similarity-based Isobaric MS2 Identification Transfer.

The identification transfer accuracy of the SIMSI pipeline was assessed by two different approaches. First, we masked 10% of the identified PSMs from each of the datasets, thus treating them as unidentified spectra from the perspective of SIMSI-Transfer. We then applied SIMSI-Transfer to the entire dataset to measure to what extent masked identifications can be recovered. For simplicity, the results of the MaxQuant analysis were considered to represent the ground truth for the purpose of this analysis. Identifications obtained by the transfer process were then compared against the ground truth data, and precision and recall were calculated (see Experimental Procedures section for details). When applying different levels of stringency (p5 to p30) in the spectrum clustering process, calculated FDRs ranged from 1.8% at the p5 setting (least stringent) to 0.2% FDR at p30 (most stringent) for the Dou *et al.* datasets (Fig. 2) The results for the phosphoproteome showed similar FDR characteristics ranging from 1.5% for p5 and 0.2% for p30. At the same time, a higher clustering stringency led to the formation of fewer clusters containing more than one spectrum. In turn, this reduces the number of transferable identifications and results in a lower recall. In addition, at the lowest clustering stringency (p5), more ambiguous clusters *(i.e.*, clusters containing spectra that represent more than one peptide) are formed, which cannot be used for transferring identifications and, therefore, also result in a lower recall. For the full proteome samples, the best recall of 85% of the masked identifications was reached at a clustering stringency of p15, and the best recall of 81% was obtained for the phosphoproteome at a stringency of p10. A precision of >99% could be obtained for clustering stringency of p10 or higher for both data types. Other masking percentages (5%, 20%, and 50%) or other datasets showed very similar trends (supplemental Fig. S1 and supplemental Tables S1–S3).

**Fig. 3:**
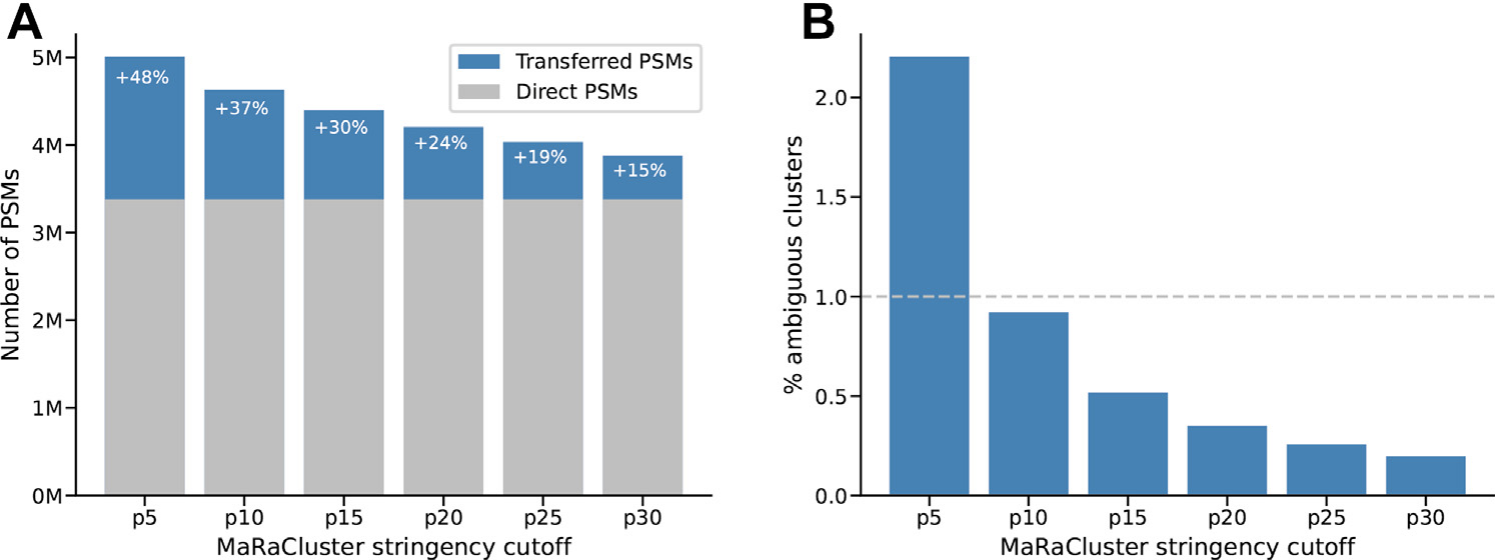
**Impact of the applied clustering threshold on identification gain and false positives.***A*, impact of the applied clustering threshold on the number of identification transfers. Identifications made by the search engine are shown in *gray*, and transferred identifications are shown in *blue*. *B*, impact of the applied clustering threshold on the percentage of ambiguous clusters (false positives). The *dotted line* marks the 1% false-positive level.

Second, we analyzed to what extent different clustering stringency settings led to ambiguous clusters, which may be considered false positives. Again, this is illustrated on the Dou *et al.* full proteome dataset, but now without masking (Fig. 3). As one might expect, the applied clustering threshold impacts the number of identifications that are transferred. More specifically, 1.6 million transfers were made for p5 (+48%) but only 0.5 million for p30 (+15%). In addition, higher clustering stringencies increased the number of clusters that contain only a single spectrum (singleton clusters). Specifically, while only 9% of all spectra remained unclustered at p5, 49% were not clustered at p30 (supplemental Figs. S2–S4). Singleton clusters cannot contribute to transferring identifications. Consequently, the increase in transferred identifications is lowest at the highest clustering stringency. Lower stringencies resulted in more transfers at the cost of more ambiguous clusters. Such ambiguous clusters are considered false positives and should, therefore, be minimized. This is illustrated in Figure 3*B*: at p5, 2.2% of all clusters of size >1 were ambiguous, 0.9% were ambiguous at p10, and 0.2% were ambiguous at p30. Interestingly, the percentage of ambiguous clusters at the different clustering stringencies was well in line with the FDR values determined from the masking analysis. As the percentage of ambiguous clusters can be determined without the need for a masking analysis, we propose that the former may be used as a proxy for estimating the FDR of identifications transferred by SIMSI-Transfer. The analogous evaluation of the other five datasets again showed very similar results (supplemental Table S4 and supplemental Figs. S2–S4). As the stringency threshold of p10 consistently resulted in FDR values of below 1% (from the masking analysis) and less than 1% ambiguous clusters, p10 was set as the default parameter for SIMSI-Transfer, and all the following analyses were performed using the p10 threshold.

### Application of SIMSI-Transfer to the Reanalysis of Six CPTAC Datasets

**Fig. 4:**
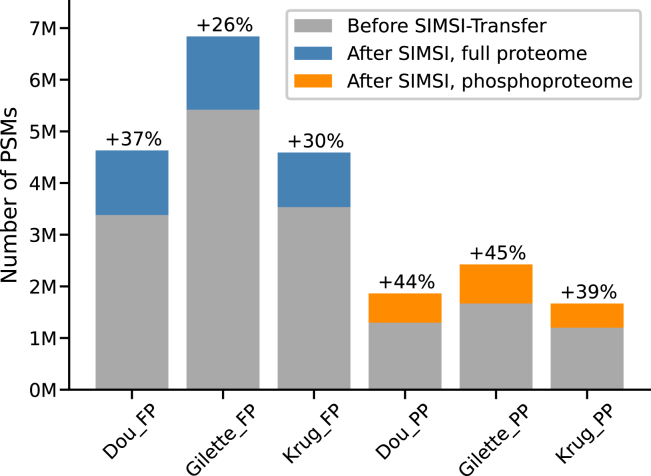
**Bar plot comparing the number of peptide-spectrum matches (PSMs) before and after applying SIMSI-Transfer in several data sets (clustering stringency p10).** SIMSI-Transfer, Similarity-based Isobaric MS2 Identification Transfer.

Figure 4 shows the results of applying SIMSI-Transfer to the datasets summarized in Table 2. It is evident that the number of identifications across TMT batches increased substantially, ranging from 26% to 37% for the full proteome data and between 39% and 45% for the phosphoproteome data. The slightly higher relative gains for the phosphoproteome data may be attributed to the fact that modified peptides and the site of modification are often more difficult to identify than unmodified sequences by database search engines. The bias of spectrum clustering should be comparatively smaller and, therefore, spectrum clustering may be more successful in relative terms as long as the modified peptide was robustly identified in at least one TMT batch.

**Fig. 5:**
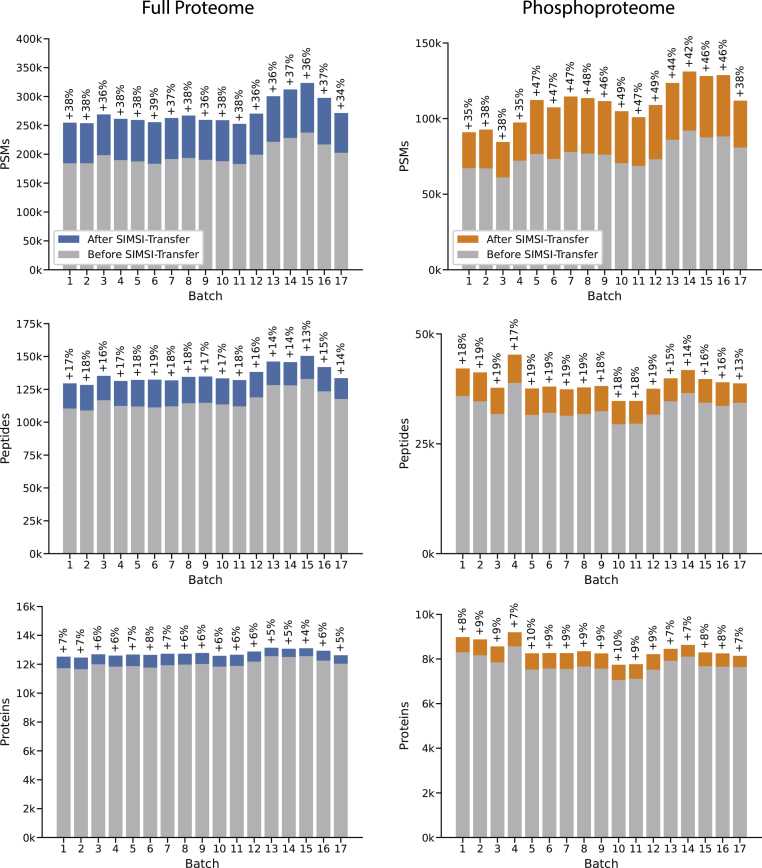
**Bar blots showing the results of applying the SIMSI-Transfer process to the Dou *et al*. dataset.** Batch-wise results are visualized for full proteome (*left*) and phosphoproteome (*right*) data at the level of PSMs (*top*), peptides (*middle*), and proteins (*bottom*). PSM, peptide-spectrum match; SIMSI-Transfer, Similarity-based Isobaric MS2 Identification Transfer.

Again exemplified on the Dou *et al.* datasets, we observed that the gains in identifications owing to the transfer process were roughly equally distributed over all batches (Fig. 5). For the full proteome data, gains ranged between 34% and 39% per batch and between 35% and 49% for the phosphoproteome data. The per-batch increase did not appear to depend on the number of PSMs within a batch. The fact that batches with fewer initial identifications also had fewer identifications after the transfer process indicates that the overall mass spectrometric data quality (*e.g.*, because of low sample loading) was different between the batches, which could not be fully compensated for by SIMSI-Transfer. At the peptide level, the gains were less pronounced but still substantial, with per-batch increases of 13 to 19% for both full proteome and phosphoproteome data. Naturally, the gains at the protein level were smaller but still a respectable 4 to 7% for the full proteome data and 7 to 10% for the phosphoproteome data. As a further plausibility check, we analyzed if the TMT ratios of the transferred identification were similar to those of the corresponding protein in the same TMT batch (supplemental Note S1). We found good agreement between these quantification values, showing that the transferred identifications generally reliably contribute to the quantification of proteins (supplemental Figs. S5–S8). In addition, we analyzed if the increase in peptide identifications with SIMSI-Transfer would have an effect on protein quantification. We analyzed a dataset with known ratios of HeLa and yeast cell lysate digests, which provided the ground truth relative quantification (supplemental Note S2). We found that while increasing the number of peptide and protein identifications per batch, the additional peptide identifications did not change protein quantification accuracy (supplemental Fig. S9).

### SIMSI-Transfer Reduces Missing Values at the Peptide and Protein Levels

**Fig. 6:**
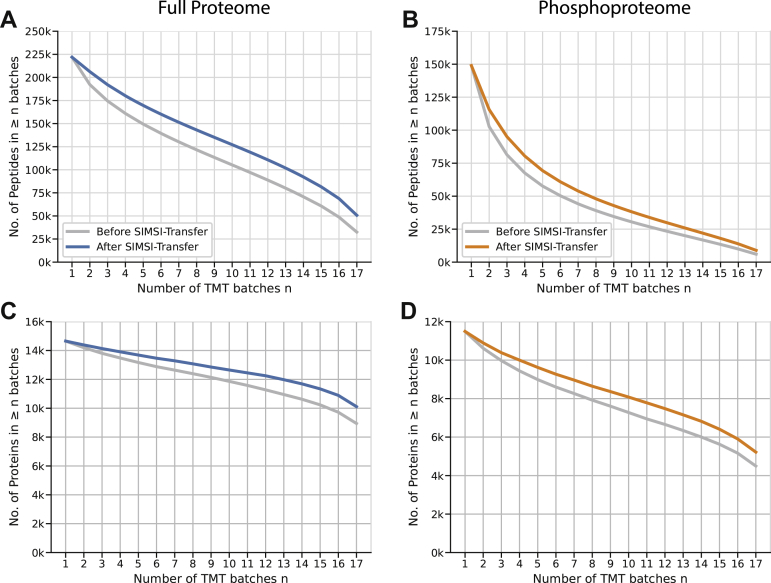
**Effect of the SIMSI-Transfer process on the number of missing peptide and protein values across TMT batches (Dou *et al.* dataset).***A*, by definition, the composite of all peptides in this study were found in at least a single batch. The number of peptides found in at least “n” batches rapidly decreases with the number of batches considered (*gray line*). Application of SIMSI-Transfer (*blue* for full proteome and *orange* for phosphoproteome) substantially improved these figures across all batches considered. SIMSI-Transfer, Similarity-based Isobaric MS2 Identification Transfer; TMT, tandem mass tag.

The occurrence of missing values when combining multiple isobarically labeled batches is a well-known phenomenon (3) and was also apparent in the data analyzed in this study (supplemental Fig. S10). In Figure 6*A*, the cumulative number of peptides found in at least *n* batches is shown before and after applying SIMSI-Transfer (Dou *et al.* dataset). Before application of SIMSI-Transfer, 113 k of 222 k peptides (51%) were found in at least half of all batches (9 of 17 batches) and only 33 k (15%) in all 17 batches. At the protein level, 12 k of 15 k (84%) were found in at least half of the batches, and 9 k (63%) were identified in all batches (Fig. 6*C*). SIMSI-Transfer increased the number of peptides found in at least *n* batches by about 20 k on average. The number of peptides found in at least half of the batches increased by 19% from 113 k to 135 k peptides, whereas the number of peptides found in all batches increased by 56% from 33 k to 51 k peptides. Similarly, the number of proteins found in all batches increased by 13% from 8.9 k to 10 k, which corresponds to a missing value reduction of 21%. For the phosphoproteome data, a total of 150 k peptides were identified in at least one batch, and only 8 k (11%) of all peptides were identified in all batches. SIMSI-Transfer increased the number of peptides found in at least half of the batches by 22% (29 k to 35 k peptides), and the number of peptides found in all 17 batches by 43% (9 k to 12 k peptides). At the protein level, 10% more proteins were found in at least half of the batches (7.6 k *versus* 8.4 k proteins) and 16% more proteins in all batches (4.5 k *versus* 5.2 k). This corresponds to a missing value reduction of 10%.

### Comparison of SIMSI-Transfer to the IMBR Function of MaxQuant

**Fig. 7:**
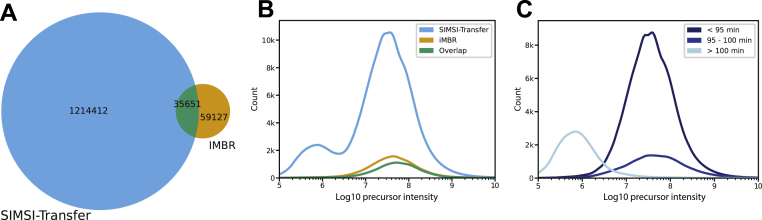
**SIMSI-Transfer outperforms the IMBR method of MaxQuant.***A*, Venn diagram showing the comparison between identifications made by SIMSI-Transfer only (*blue*), by IMBR only (*orange*), and by both (*green*). *B*, intensity distribution of precursor ions identified by SIMSI-Transfer only (*blue*), by IMBR only (*orange*), and by both (*green*). *C*, precursor ion intensity distribution of all SIMSI-Transfer identifications split by chromatographic elution time. IMBR, isobaric match-between-runs; SIMSI-Transfer, Similarity-based Isobaric MS2 Identification Transfer.

The MaxQuant software comes with the IMBR function that attempts to transfer peptide identifications in isobaric labeling data. To compare IMBR to SIMSI-Transfer, two MaxQuant analyses were performed on the Dou *et al.* dataset: one with and one without IMBR enabled. The results without IMBR were used as input for SIMSI-Transfer and compared with results obtained by IMBR. Enabling IMBR led to the identification of an additional 94 k (+2.8%) spectra with TMT quantifications. In stark contrast, applying SIMSI-Transfer yielded 1.3 million (+37%) transferred identifications. The overlap between IMBR and SIMSI-Transfer was 36 k transferred identifications, corresponding to 38% of the total number of transfers made by IMBR (Fig. 7*A*). The limited overlap between the results of the two algorithms is surprising, particularly because the majority of cases that are unique to IMBR, unique to SIMSI-Transfer, or shared between the two represent high-intensity precursor ions (Fig. 7*B*).

We assessed the discrepancy between the two approaches by analyzing spectra identified exclusively by one of the two tools. For the spectra uniquely identified by SIMSI-Transfer, we found that ∼30% did not have a precursor assigned in the MaxQuant output files, which excludes them from consideration by MBR. Furthermore, we found that the standard deviation of retention times for each peptide across multiple batches was far larger than the default retention time matching tolerance of 0.7 min, even after MaxQuant’s retention time calibration step (supplemental Fig. S11). While one could increase this tolerance, this would further exacerbate the false matching problem of MBR (8). Conversely, we found that spectra identified by IMBR but not by SIMSI-Transfer often exhibited low similarity to the *in silico* predicted fragment spectrum of the peptide sequence by Prosit (© Chair of Proteomics and Bioanalytics) (24) (supplemental Fig. S12), making such identifications questionable for use in peptide and protein quantification. This is a result of IMBR exclusively relying on MS1 feature matching, subject to the abovementioned false matching problem, whereas SIMSI-Transfer uses MS2 spectrum similarity. The exact reason for the small overlap between the two approaches remains to be investigated in more detail in the future.

Another interesting observation for SIMSI-Transfer was a bimodal distribution consisting of (i) high-intensity precursors identified across the acetonitrile gradient of the LC-MS/MS run, and (ii) lower-intensity precursors eluted at high concentrations of organic solvent used for column washing at the end of the run (Fig. 7*C*). While MaxQuant and IMBR struggled to identify those precursors, SIMSI-Transfer identified many spectra from low-abundance precursors in this area. To ascertain that these low-abundance precursor identifications are trustworthy, we again resorted to comparing TMT reporter ion intensities between PSMs made by MaxQuant or transferred by SIMSI-Transfer to those of their corresponding proteins in the same TMT batch (supplemental Note S1 and supplemental Fig. S13). There was very good correspondence, which implies that the transferred identifications are as reliable for protein quantification as identifications directly made by MaxQuant.

## Discussion

The results presented in this study demonstrate that the SIMSI-Transfer pipeline improves the number of MS2 spectra that lead to peptide identifications in isobaric labeling experiments. It also reduces missing values at the level of PSMs, peptides, and proteins when combining multiple TMT batches, and this was true for both proteome and phosphoproteome data. Both aspects are relevant for large-scale isobaric labeling studies in which many samples are distributed over many TMT batches, as illustrated by the reanalysis of a number of clinical cohorts from the CPTAC project. Although not tested here specifically, the improved data consistency will facilitate a more consistent recognition and comparison of cancer-relevant proteins and pathways and, more generally, an increase in statistical power.

Two independent methods were used to assess the quality of SIMSI-Transfer results. The masking analyses allowed computing an FDR, and calculating the number of ambiguous clusters turned out to be a good proxy for FDR as the clustering thresholds required for both approaches were the same. At the recommended threshold of p10, both FDR and the percentage of ambiguous clusters were below 1%. We cannot guarantee that this threshold is optimal for every dataset, but we encourage users to explore the effects of different thresholds on their respective data using the software provided in this article.

SIMSI-Transfer strongly outperformed the IMBR function of MaxQuant, but the overlap between the two approaches was surprisingly low. The reasons for this discrepancy remain elusive at present. In addition, we note that we were unable to reproduce the results reported in the original IMBR publication, on the dataset used in the original publication (data not shown) (13). As an alternative to MBR, Corthésy *et al.* (25) have shown substantial gains in identifications on cerebrospinal fluid samples using the similarity of MS2 spectra to consensus spectra. However, this project is no longer maintained, and we were unable to get this tool to work.

Distinguishing between different phosphorylation sites within a peptide sequence, especially if the potential phosphorylation sites are in close proximity, remains challenging for SIMSI-Transfer. This is not an exclusive shortcoming of the presented method but rather a general issue in peptide identification/localization by classical database searching. When only a few (if any) fragment ions can be observed in MS2 spectra that distinguish the different localization possibilities, it becomes increasingly more difficult to locate the modification site accurately (26). It should be noted that database search algorithms such as Andromeda from MaxQuant and Mascot by Matrix Science also suffer from this issue (27), and other more dedicated software packages can be used to resolve such ambiguities at least partially (28, 29, 30, 31). We chose to retain such ambiguous phosphopeptide spectra clusters because we deem these to contain valuable information for further biology-centered data analysis. For now, the user will be informed about localization ambiguity, and extending SIMSI-Transfer to resolve ambiguities of PTM-isomeric peptides will be investigated in future work.

SIMSI-Transfer provides a graphical user interface but can also be run from the command line. As input, it only requires the MaxQuant output folder as well as the ∗.raw files from the mass spectrometer, which get converted to ∗.mzML files internally using the TermoRawFileParser by CompOmics (32). As a proxy for the FDR of the transferred identifications, SIMSI-Transfer determines the number of ambiguous clusters in the run, which closely followed the FDR calculated in the masking analysis. Optionally, an FDR can be calculated using the masking approach for higher confidence. The user can run multiple clustering thresholds to select the appropriate stringency for further downstream analysis. SIMSI-Transfer can be used to handle large cohort studies. The largest dataset analyzed here comprised 625 raw files and was processed on a desktop personal computer in less than 1 day.

Further improvements of SIMSI-Transfer can be envisaged. Currently, it uses MaRaCluster for MS2 spectrum clustering in conjunction with identification results from MaxQuant but can be extended to handle outputs from other database search engines. The integration of other clustering tools can also be considered, but prior work indicated that, for LFQ data, different tools yield comparable results (16). SIMSI-Transfer is an open-source software. Its modular design enables its integration into other established data processing pipelines. The authors, therefore, anticipate that SIMSI-Transfer constitutes a useful tool for the scientific community for the integration of large-scale proteomic studies that use isobaric stable isotope labeling strategies.

## Data Availability

All raw MS data used in this article can be downloaded from the CPTAC data portal at https://proteomic.datacommons.cancer.gov/pdc/browse (cohort datasets) or *via* PRIDE (Thompson *et al.* mixture dataset). SIMSI-Transfer is available on GitHub (https://github.com/kusterlab/SIMSI-Transfer), and all files generated during our experiments can be found on zenodo.org *via* the DOIs 10.5281/zenodo.6365902 (MaxQuant output) and 10.5281/zenodo.6365638 (SIMSI-Transfer output).

## Supplemental data

This article contains supplemental data.

## Conflict of interest

The authors declare no competing interests.

## Supplemental Data

SIMSI-Transfer_supplement
