# Supplementary material for: SIMSI-Transfer: Software-Assisted Reduction of Missing Values in Phosphoproteomic and Proteomic Isobaric Labeling Data Using Tandem Mass Spectrum Clustering
Source: Mol Cell Proteomics. 2022 Apr 21;21(8):100238. doi: 10.1016/j.mcpro.2022.100238 (PMC9389303; doi:10.1016/j.mcpro.2022.100238)
Supplement: SIMSI-Transfer_supplement [file mmc1.docx]

SIMSI-Transfer paper – Supplementary materials


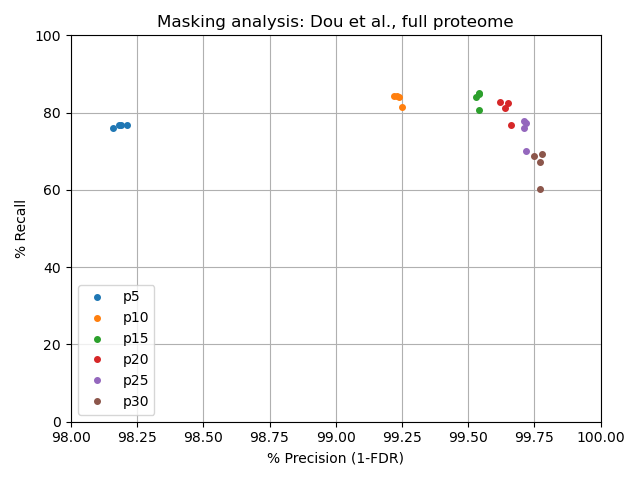

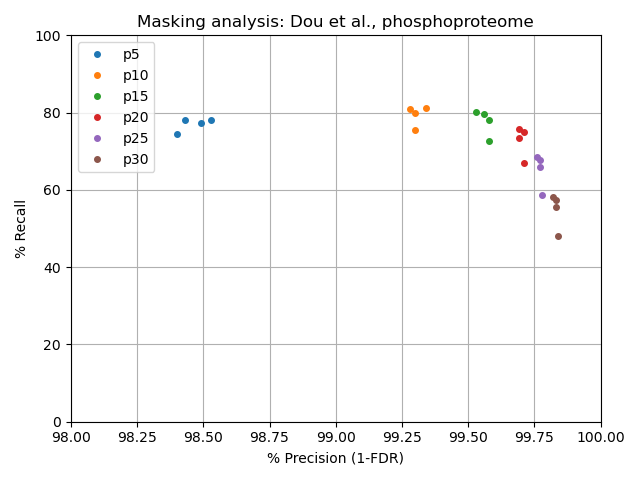


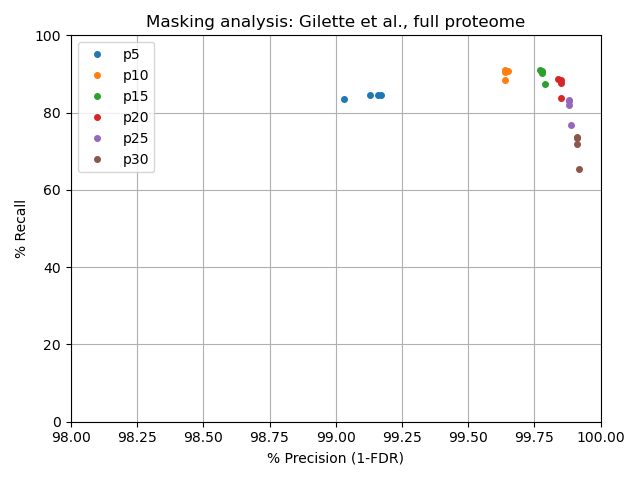

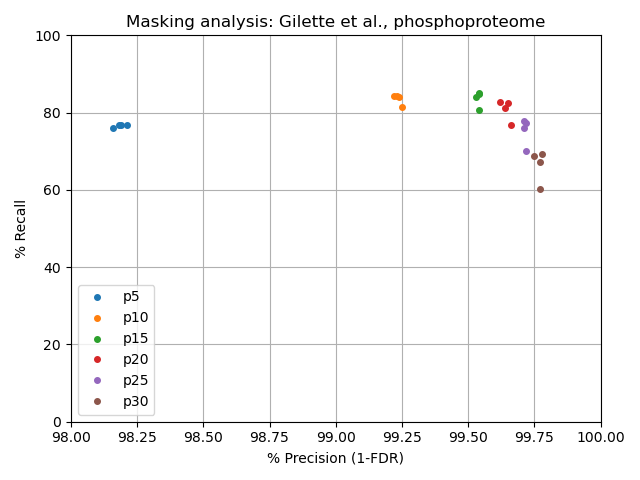


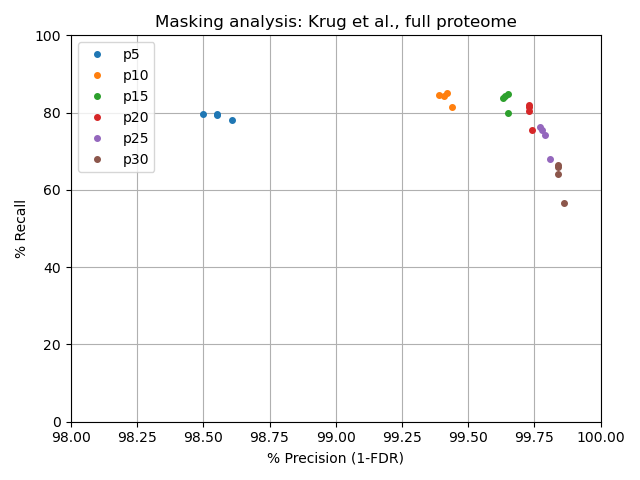

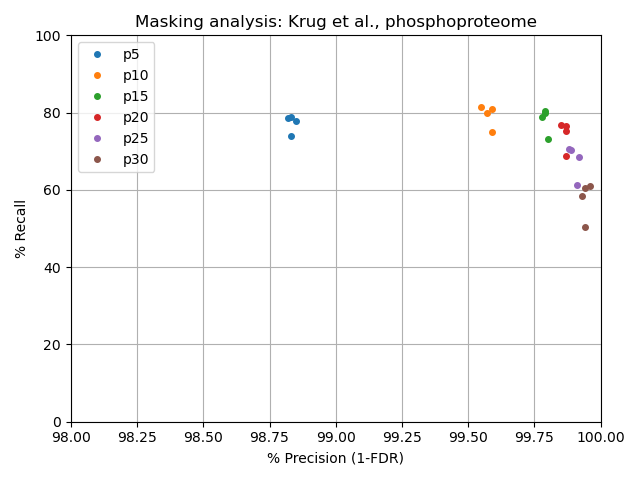


Supplementary figure 1: Precision-recall-plots for the masking analysis of all six datasets. Each group of points consists of the four masking percentages 5%, 10%, 20%, and 50%.

Supplementary table 1: Masking analysis recall comparison for different masking percentages; applied clustering stringency p10. A higher masking percentage reduces the number of potential transfers due to less available transfer donors, thereby decreasing the recall.

| **Recall [%]** | Dou et al. | | Gillette et al. | | Krug et al. | |
| --- | --- | --- | --- | --- | --- | --- |
| Masking | FP | PP | FP | PP | FP | PP |
| 5% | 84.4 | 81.2 | 90.9 | 86.4 | 84.9 | 81.5 |
| 10% | 84.4 | 80.9 | 90.8 | 86.1 | 84.6 | 81 |
| 20% | 83.9 | 79.9 | 90.4 | 85.3 | 84.3 | 70 |
| 50% | 81.6 | 75.6 | 88.4 | 81.6 | 81.6 | 75 |

Supplementary table 2: Masking analysis FDR comparison for different masking percentages; applied clustering stringency p10. A higher masking percentage has no significant effect on the FDR, as the correctness of transfers is less affected by the number of potential transfer donors.

| **FDR [%]** | Dou et al. | | Gillette et al. | | Krug et al. | |
| --- | --- | --- | --- | --- | --- | --- |
| Masking | FP | PP | FP | PP | FP | PP |
| 5% | 0.77 | 0.66 | 0.36 | 0.35 | 0.59 | 0.45 |
| 10% | 0.78 | 0.72 | 0.35 | 0.39 | 0.59 | 0.41 |
| 20% | 0.76 | 0.7 | 0.35 | 0.4 | 0.59 | 0.43 |
| 50% | 0.75 | 0.7 | 0.35 | 0.39 | 0.57 | 0.41 |

Supplementary table 3: Masking analysis: Recovery and FDR values of all datasets

Supplementary table 4: Clustering result summary of all datasets. The identification rate of the MaxQuant searches varies greatly between full proteome and phosphoproteome analysis, with the phosphoproteome samples generally showing a much lower identification rate.

| Study | Total MS2 spectra | Transferred IDs | Total IDs | | ID increase | ID rate | |
| --- | --- | --- | --- | --- | --- | --- | --- |
|  |  |  | w/o clustering | with clustering |  | w/o clustering | with clustering |
| Dou FP | 16.365.521 | 1.250.063 | 3.380.096 | 4.630.159 | 37,0 % | 20,7 % | 28,3 % |
| Dou PP | 8.632.824 | 568.304 | 1.294.965 | 1.863.269 | 43,9 % | 15,0 % | 21,6 % |
| Gillette FP | 24.535.656 | 1.426.088 | 5.423.670 | 6.849.758 | 26,3 % | 22,1 % | 27,9 % |
| Gillette PP | 14.142.406 | 757.003 | 1.667.262 | 2.424.265 | 45,4 % | 11,8 % | 17,1 % |
| Krug FP | 17.187.403 | 1.058.424 | 3.537.010 | 4.595.434 | 29,9 % | 20,6 % | 26,7 % |
| Krug PP | 9.959.637 | 470.078 | 1.197.787 | 1.667.865 | 39,2 % | 12 % | 16,7 % |

Dou et al.

Full Proteome Phosphoproteome


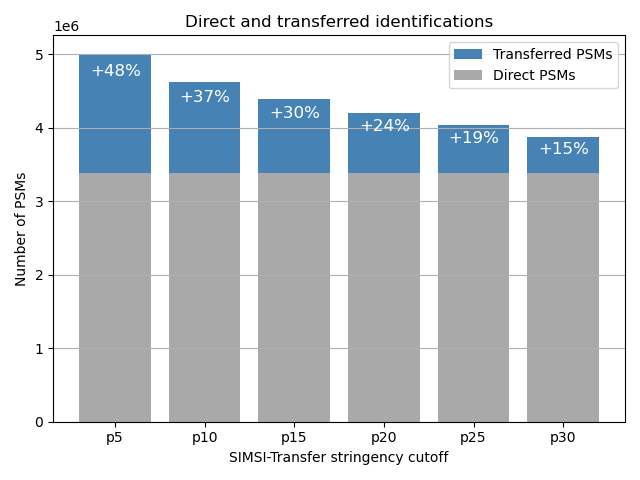

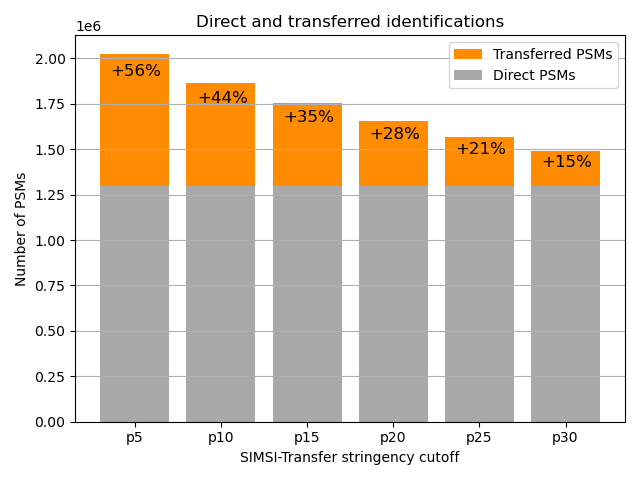


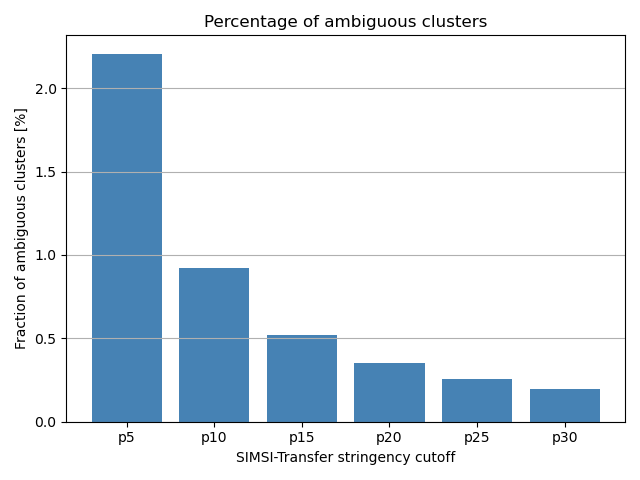

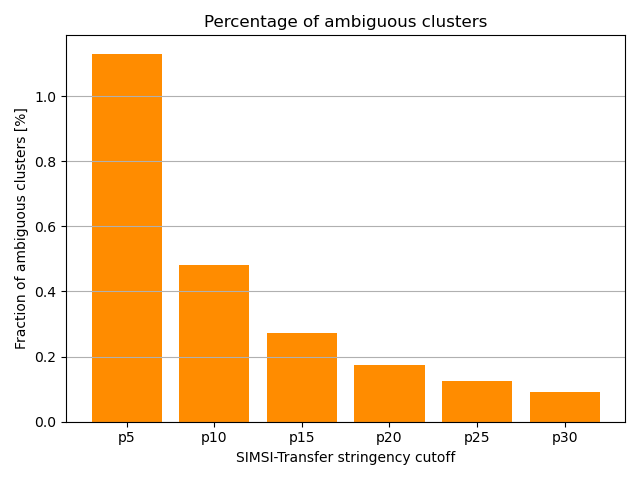


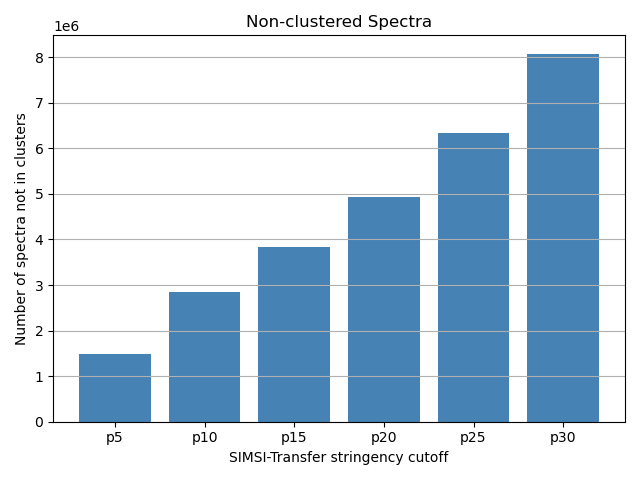

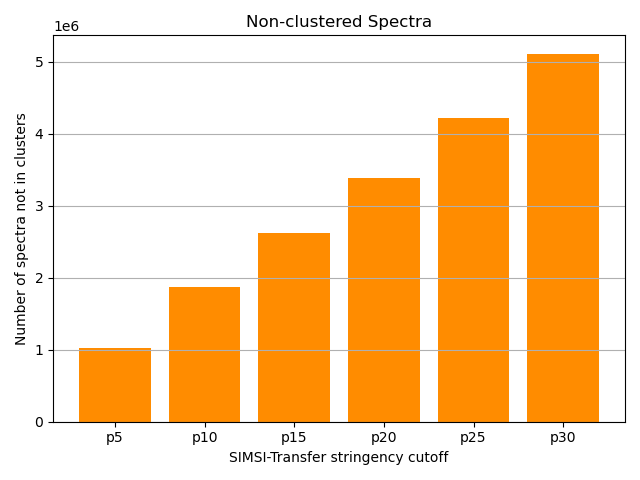


Supplementary figure 2: Identifications (top), ambiguous clusters (middle), and non-clustered spectra (bottom) for full proteome (left) and phosphoproteome (right) samples of the Dou et al. dataset

Gillette et al.

Full Proteome Phosphoproteome


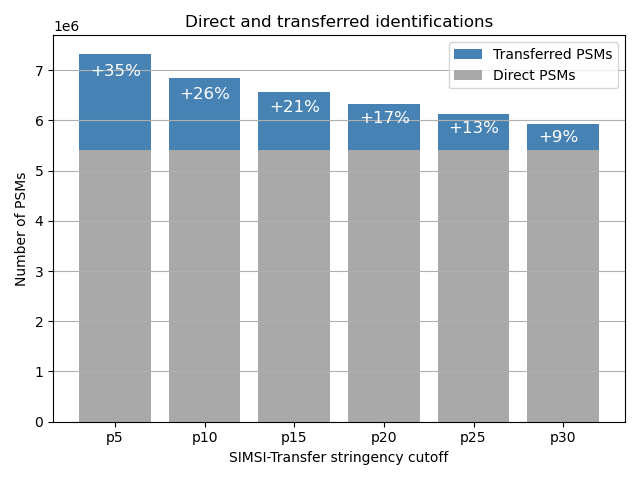

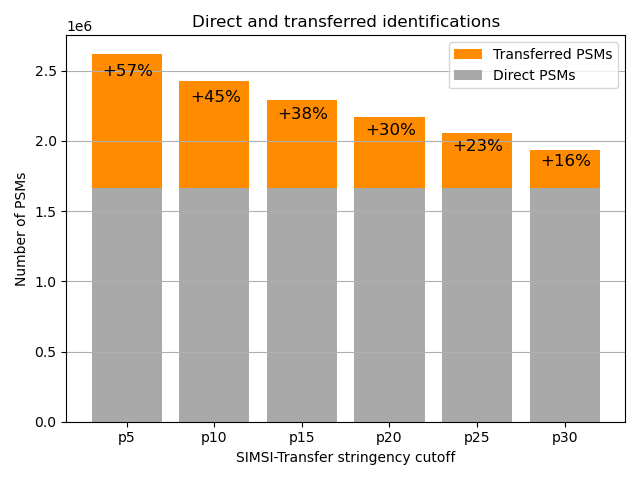


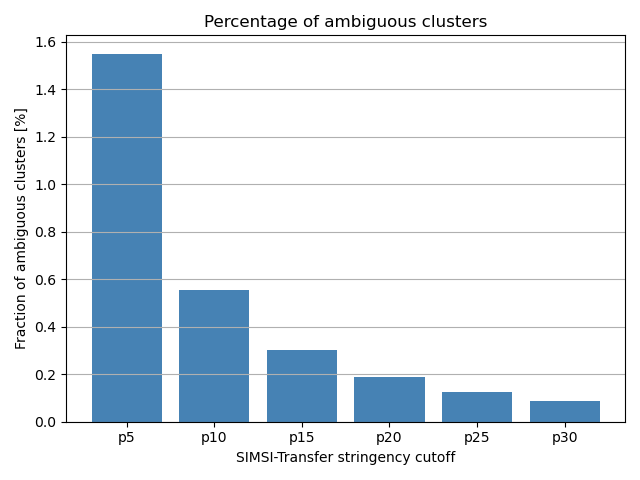

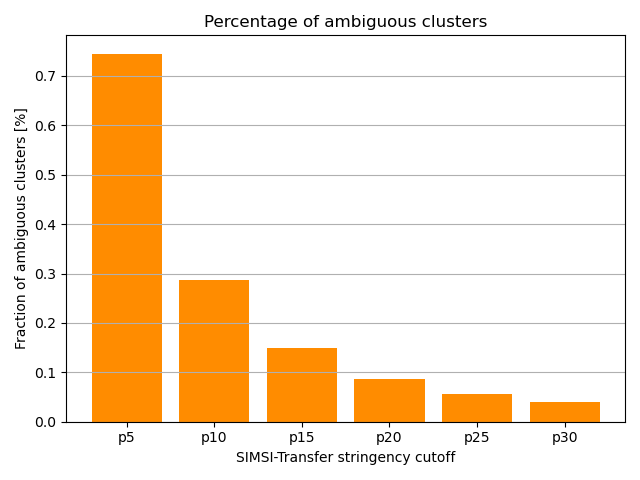


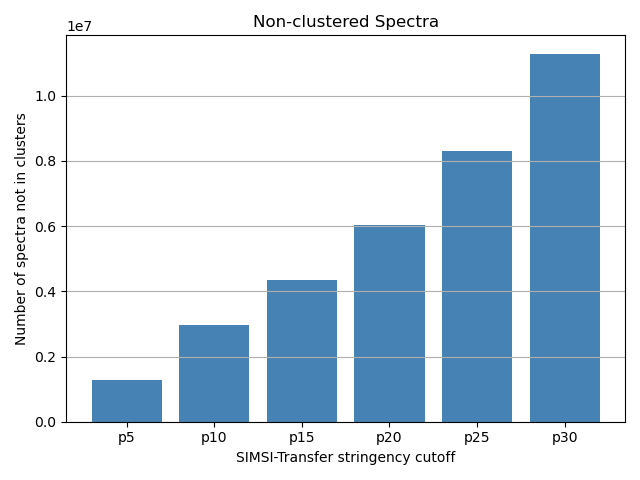

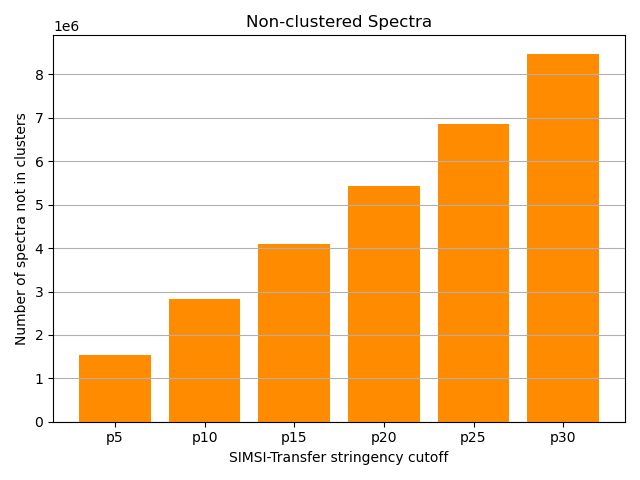


Supplementary figure 3: Analysis of the Gillette et al. dataset. Number of Identifications (top), percentage of ambiguous clusters (middle), and number of non-clustered spectra (bottom) for full proteome (left) and phosphoproteome (right).

Krug et al.

Full Proteome Phosphoproteome


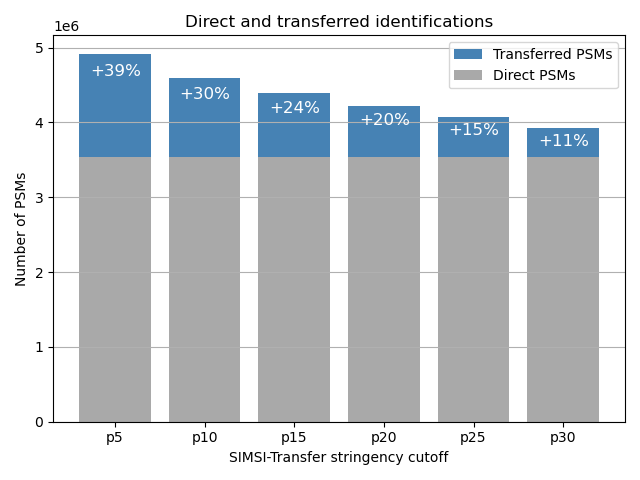

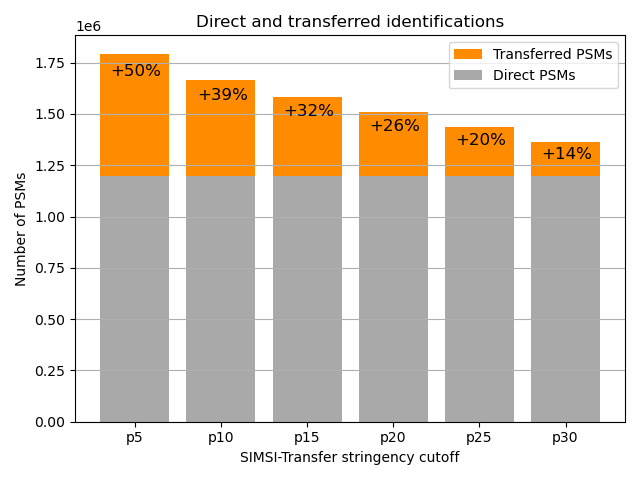


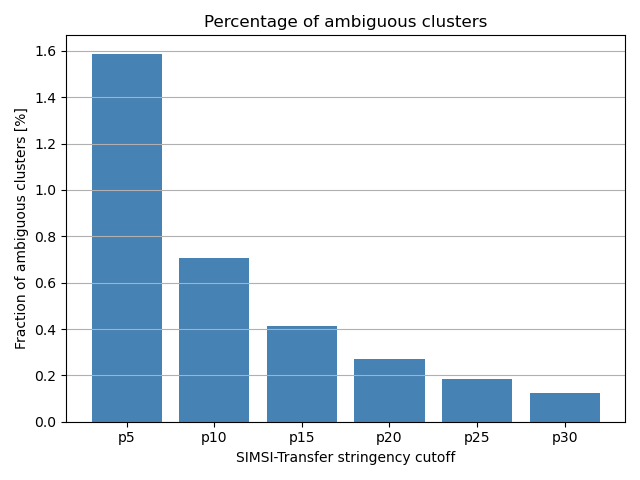

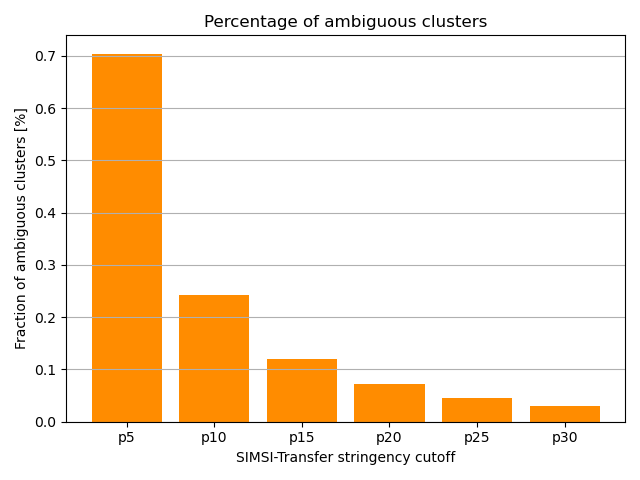


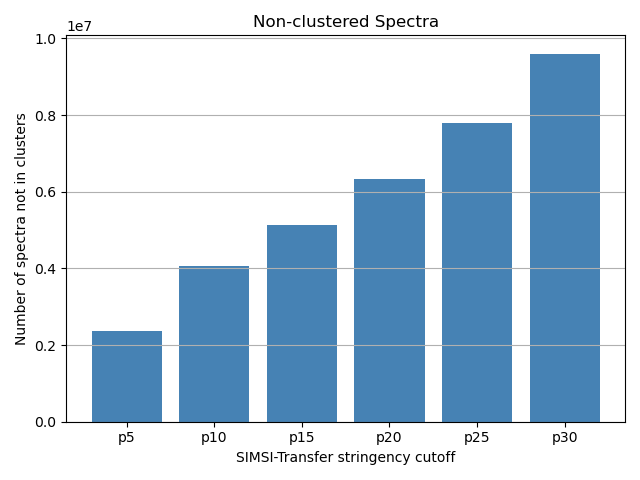

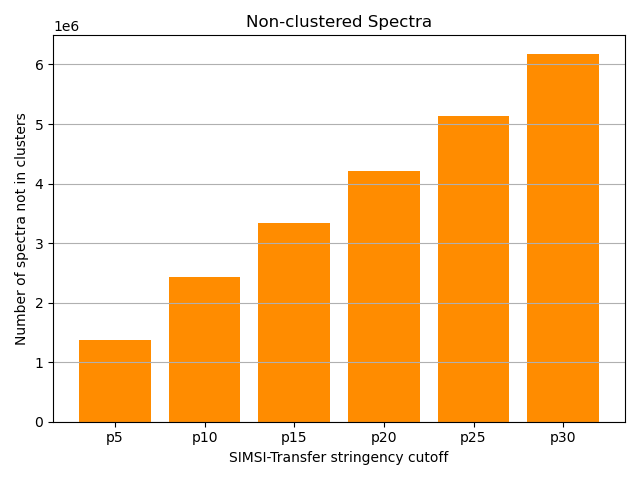


Supplementary figure 4: Analysis of the Krug et al. dataset. Number of Identifications (top), percentage of ambiguous clusters (middle), and number of non-clustered spectra (bottom) for full proteome (left) and phosphoproteome (right).

Supplementary note 1: Reporter correlation experiments Dou et al.

To assess if the transferred IDs could reliably be used for protein quantification, we used the Dou et al. dataset to test if the transferred identifications had similar TMT ratios as their corresponding protein in the same TMT batch (Supplementary figure 5, top). We calculated the TMT ratios of the protein by summing up the TMT reporter ions of all PSMs for that protein per TMT batch. For each transferred identification, we then calculated the Pearson correlation coefficient between its TMT ratios and those of its associated protein.

To put the distribution of such correlations into context, we also calculated the correlations of MaxQuant identifications to their corresponding protein, making sure to remove the TMT reporter intensities of that particular identification from the protein TMT ratio before calculating the correlation (Supplementary figure 5-8, blue). Additionally, we computed the correlation of the TMT ratios of MaxQuant identification to a randomly selected protein (yellow).

As shown in Supplementary figure 6 and 8, the Pearson correlation distributions of transferred identifications are highly similar to the distribution of MaxQuant identifications and highly dissimilar to the distribution of correlations with random proteins. Higher clustering stringencies result in higher similarity of the transferred distribution to the MaxQuant distribution.


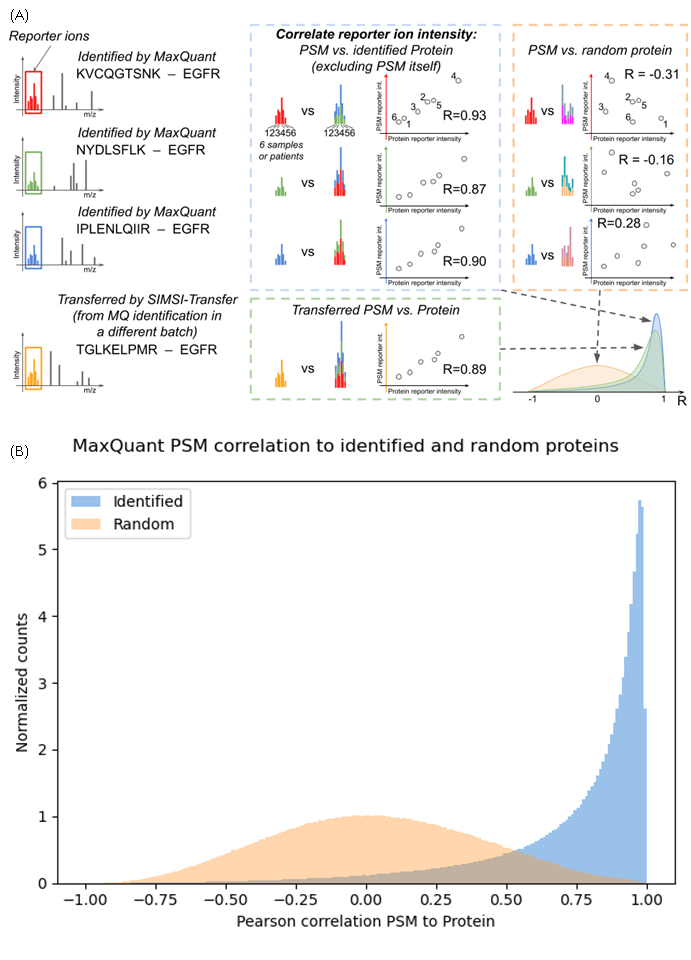


Supplementary figure 5: (A) Overview of reporter ion correlation experiments. We compute Pearson correlations between a spectrum’s TMT ratios and its corresponding protein.
(B) TMT reporter correlation distributions of full proteome spectra identified by MaxQuant to their allocated protein (blue) and to random proteins (orange)


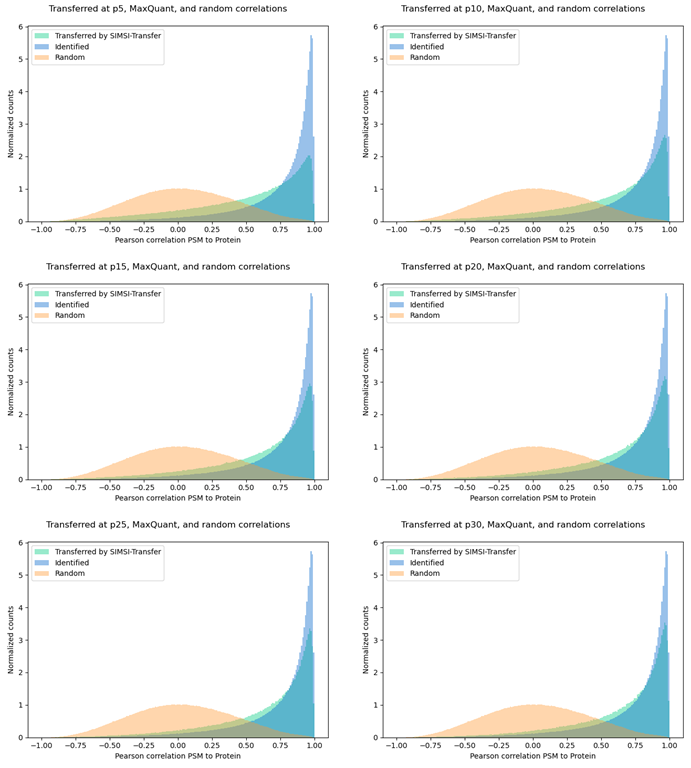


Supplementary figure 6: TMT reporter correlation distributions of full proteome spectra identified by SIMSI-Transfer to their allocated protein (green); the distributions show high similarity to the correlation distribution generated by MaxQuant IDs (blue), and a low similarity with correlations to random proteins (orange). In addition, a higher clustering stringency causes this similarity to further increase, as less false positives with low correlation values are generated.


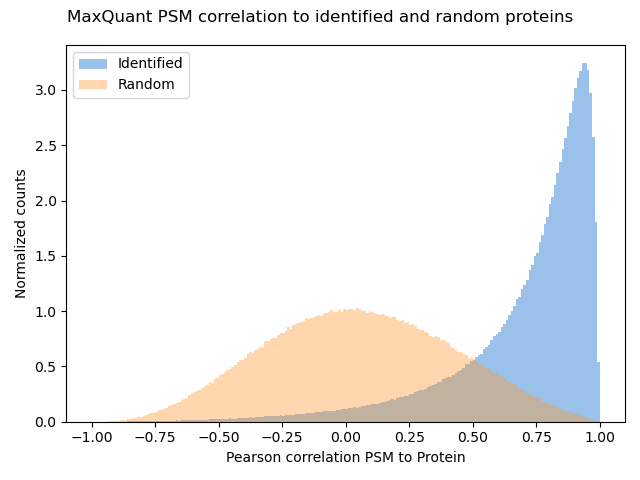


Supplementary figure 7: TMT reporter correlation distributions of phosphoproteome spectra identified by MaxQuant to their allocated protein (blue) and to random proteins (orange)


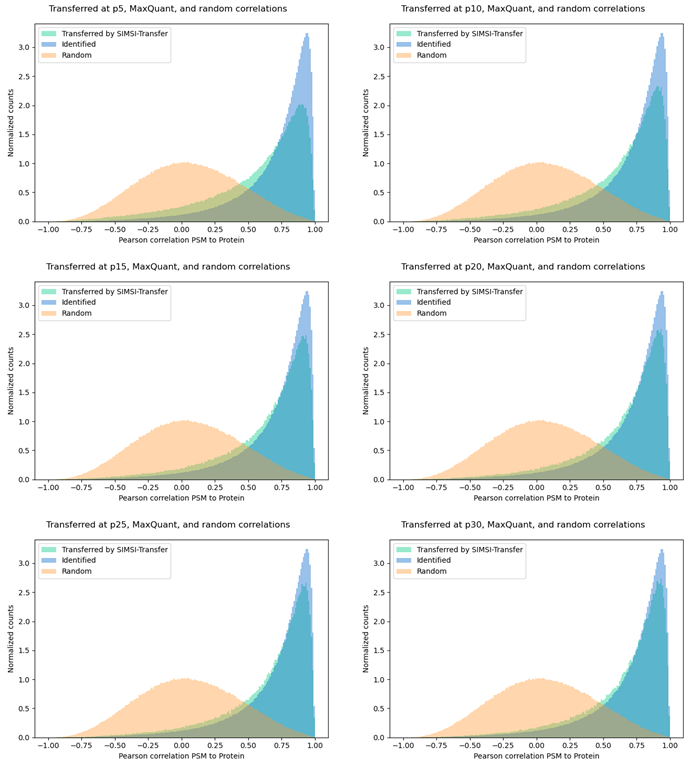


Supplementary figure 8: TMT reporter correlation distributions of phosphoproteome spectra identified by SIMSI-Transfer to their allocated protein; similar to the full proteome distributions, the phosphoproteome distributions show high similarity to the correlation distribution generated by MaxQuant IDs (blue), and a low similarity with correlations to random proteins (orange). A higher clustering stringency causes a further increase in similarity to the MaxQuant correlation distribution.

Supplementary Note 2: Effect of SIMSI-Transfer on quantification accuracy

To assess whether the PSMs gained by applying SIMSI-Transfer affect peptide- or protein-level quantifications, we used the Thompson et al. dataset. It consists of three TMT batches with known ratios of yeast and HeLa lysates in each batch. We calculated the average ratio pattern for each peptide and protein and compared them to the ground truth ratios both before and after applying SIMSI-Transfer (Supplementary figure 9). Overall, the additional identifications gained by SIMSI-Transfer showed highly similar reporter intensities compared to the MaxQuant identifications, and therefore the additional identifications only had a minor effect on the quantifications.


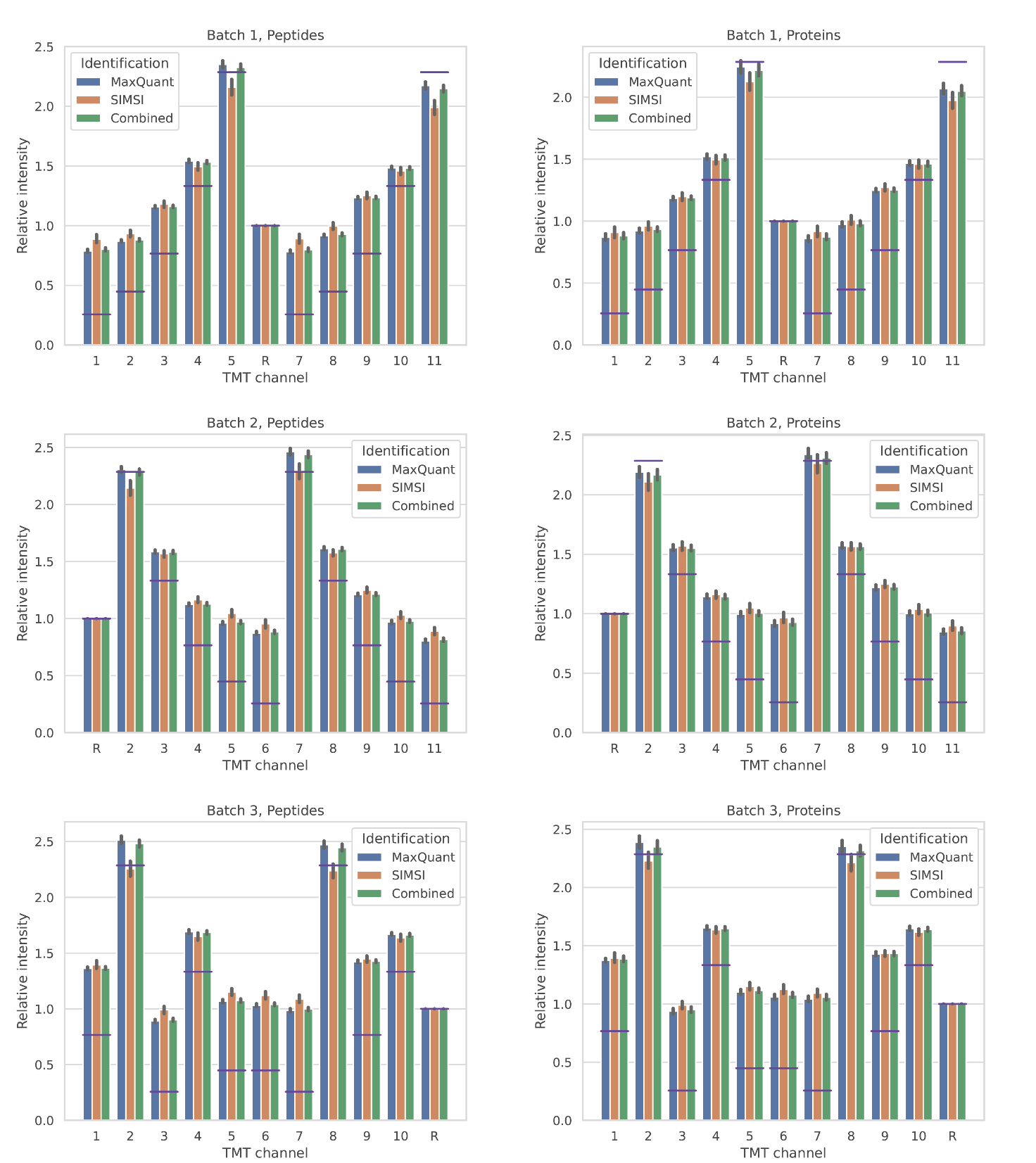


Supplementary figure 9: Comparison of peptide (left) and protein (right) TMT reporter pattern to ground truth values (violet lines). MaxQuant identifications (blue) show similar trends as the ground truth ratios, but the measured ratios are affected by a strong ratio compression due to chimeric spectra. The reporter intensities of SIMSI-Transfer identifications (beige) as well as the combined reporter intensities of all identifications (green) show minor shifts, but overall look highly similar to the pattern observed with MaxQuant only. The additional PSMs increase the number of peptides obtained in the experiment while not impacting the peptide-level quantification in a negative manner.

**Inflation of missing values when combining TMT batches**


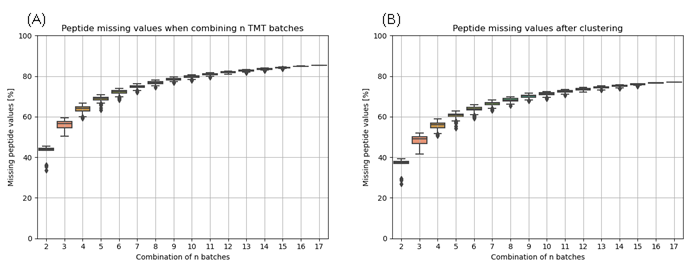


Supplementary figure 10: Combining multiple batches of TMT experiments from the Dou et al. dataset leads to missing values, depending on the number of batches combined. (A) Before clustering, about 40% missing values are observed for the combination of any two batches, increasing to 85% missing values when combining all batches. (B) After clustering, the missing values show a similar course, however the values are decreased by 6 – 8 percentage points.


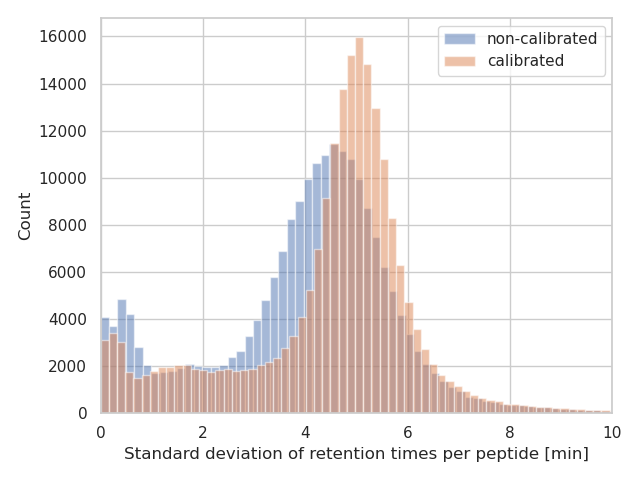


Supplementary figure 11: Standard deviations of retention time for each peptide across batches. The uncalibrated retention time (blue) shows a peak at standard deviations of around 4.5 minutes, which increases to 5.1 minutes after calibration (orange) rather than decreasing. This is much larger than the 0.7 retention time tolerance window used for Isobaric Match Between Runs (IMBR) and could explain why SIMSI-Transfer leads to many more identification transfers than IMBR, as SIMSI-Transfer does not use a retention time tolerance window.


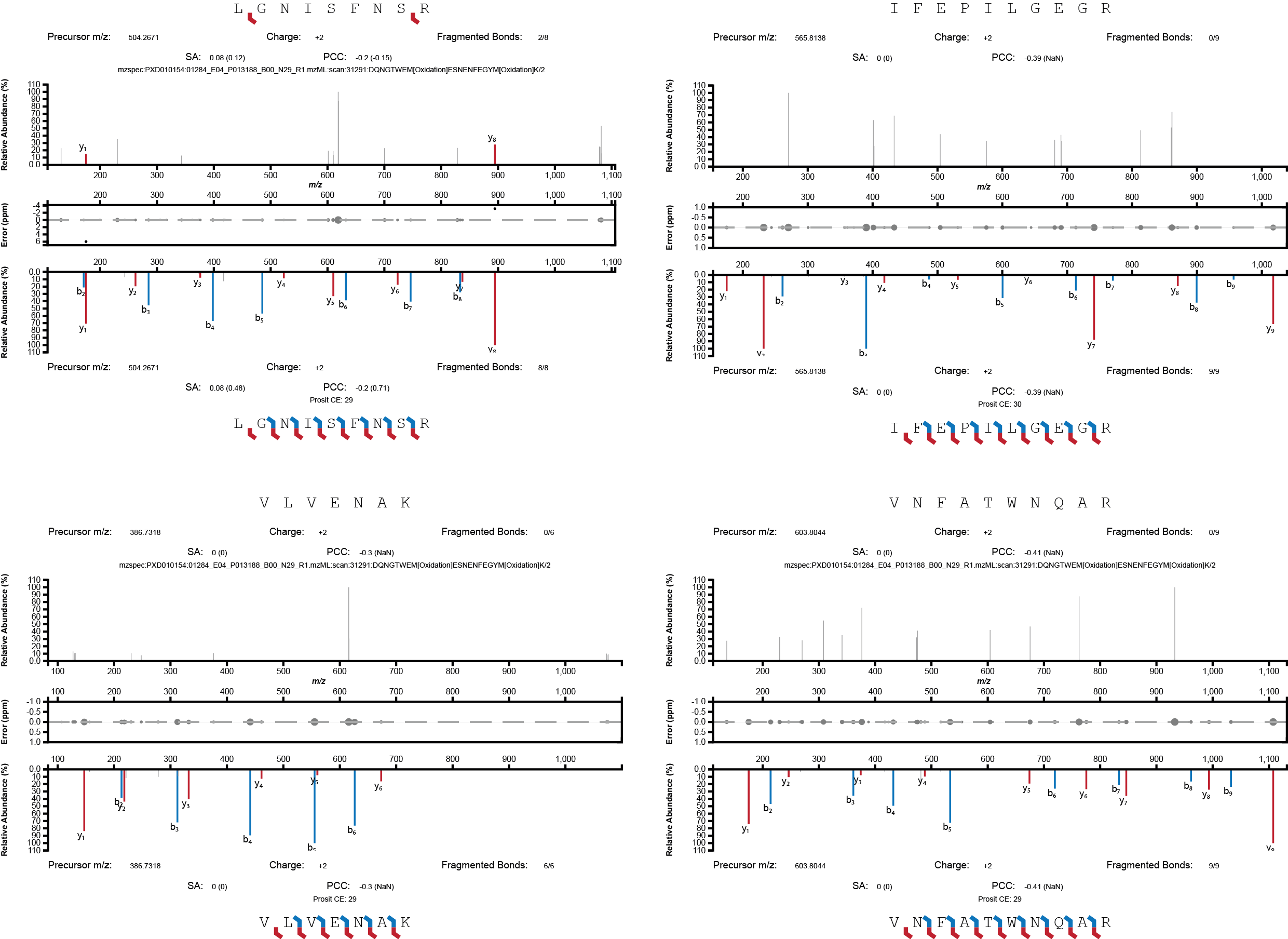


Supplementary figure 12: Example mirror plots of match between runs matches. MS2 spectrum identified by Isobaric Match between Runs (IMBR, top) and predicted MS2 spectrum by PROSIT (bottom). As IMBR does not compare MS2 spectra while performing the matching, it identifies spectra of lower quality and more noise. Identification transfers to spectra like the ones shown here are far less likely to occur for SIMSI-Transfer because it relies on MS2 spectrum similarity rather than MS1 feature alignment.


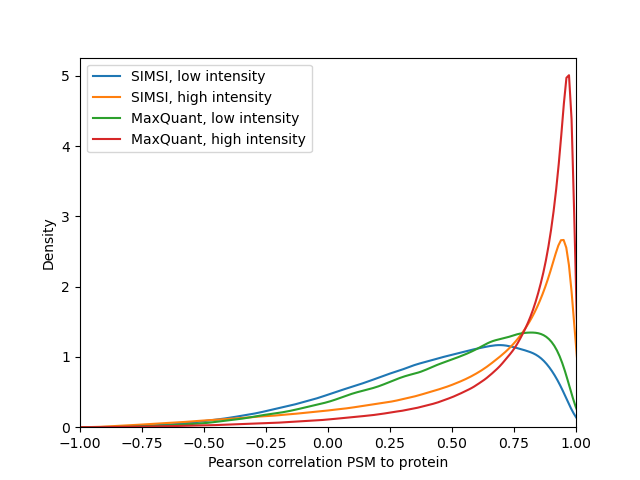


Supplementary figure 13: Pearson correlation distribution (see Supplementary Note 1) of PSMs obtained by MaxQuant and by SIMSI to their proteins, split by intensity. MaxQuant and SIMSI-Transfer PSMs show highly similar distributions in their respective intensity ranges.
